# Temperature-Dependent Reorganization of Conformational Dynamics and Interaction Networks Underlies Thermostability in PET-Degrading Enzymes

**DOI:** 10.3390/ijms27146531

**Published:** 2026-07-22

**Authors:** Hui Duan, Chen Wan, Bu-Qing Wang, De-Rui Zhao, Meng-Ting Liu, Li-Quan Yang, Peng Sang

**Affiliations:** 1 College of Agriculture and Biological Science, Dali University, Dali 671000, China; dhui4399@gmail.com (H.D.); wc4082@gmail.com (C.W.); 15393947279@163.com (B.-Q.W.); zderay945@gmail.com (D.-R.Z.); lminimt@163.com (M.-T.L.); 2 Key Laboratory of Bioinformatics and Computational Biology of the Department of Education of Yunnan Province, Dali University, Dali 671000, China; 3 Co-Innovation Center for Cangshan Mountain and Erhai Lake Integrated Protection and Green Development of Yunnan Province, Dali University, Dali 671000, China

**Keywords:** polyethylene terephthalate, PETase, thermostability, molecular dynamics simulations, neural relational inference, enzyme engineering

## Abstract

Polyethylene terephthalate (PET) is one of the most widely used synthetic plastics and a major contributor to global plastic pollution because of its high resistance to degradation. Enzymatic degradation by PET hydrolases (PETase) has emerged as a sustainable strategy for PET recycling; however, the limited thermostability of wild-type PETase restricts its industrial application. To elucidate the molecular basis underlying the different thermal behaviors of PET hydrolases, long-timescale molecular dynamics simulations were performed on WT-PETase, FAST-PETase, and the thermostable cutinase variant LCC-ICCG at 30 °C, 50 °C, and 70 °C. Comparative analyses integrating structural stability, residue flexibility, rigidity networks, free energy landscapes, and neural relational inference models revealed that FAST-PETase and LCC-ICCG exhibited enhanced conformational stability and reduced structural flexibility compared with WT-PETase, particularly under elevated temperatures. The improved thermostability was associated with more compact free energy landscapes, strengthened residue interaction networks, and better preservation of the catalytic architecture during thermal perturbation. These results suggest that an optimal balance between structural rigidity and conformational flexibility is critical for maintaining enzyme stability at elevated temperatures. Overall, this study provides molecular-level insights into the structural determinants of PETase thermostability and offers a theoretical framework for the rational engineering of efficient and heat-resistant plastic-degrading enzymes.

## 1. Introduction

Polyethylene terephthalate (PET) is one of the most widely produced synthetic polymers and a major contributor to persistent plastic pollution [1]. Enzymatic depolymerization has emerged as a promising strategy for sustainable PET recycling owing to its chemical selectivity, low environmental footprint, and compatibility with circular-economy concepts [2,3]. However, the feasibility of enzymatic PET degradation is fundamentally constrained by the physical properties of the polymer substrate itself [4,5]. As a semi-crystalline material, PET exhibits limited chain mobility at ambient temperatures, and efficient hydrolysis becomes kinetically accessible only near its glass transition temperature (65–75 °C) [6,7], where segmental motions are sufficiently activated to expose ester bonds embedded within the solid matrix. Consequently, effective PET depolymerization requires enzymes to operate under elevated thermal conditions dictated not by enzymatic preference, but by polymer physics [8,9].

This substrate-imposed temperature requirement places a stringent and unavoidable constraint on biocatalysts. To be practically effective, enzymes must remain functionally competent precisely within the thermal regime that maximizes PET chain mobility [10,11,12]. Yet most PET-degrading enzymes identified to date are mesophilic, exhibiting optimal activity at moderate temperatures while undergoing rapid thermal deactivation upon heating [13,14,15]. Wild-type PETase exemplifies this incompatibility, displaying appreciable activity at 30–50 °C but losing catalytic competence well below PET’s glass transition temperature [16,17]. Importantly, this mismatch is not limited to a single enzyme variant but reflects a broader challenge shared across PET hydrolases isolated from diverse biological sources [18,19,20]. As a result, the central bottleneck of enzymatic PET recycling is not simply catalytic efficiency, but the ability of enzymes to preserve functional conformational states under sustained thermal stress [21].

To address this limitation, extensive protein-engineering efforts have focused on enhancing the thermal stability of PET-degrading enzymes [22,23]. Structure-guided mutagenesis, consensus-based design, and data-driven optimization strategies have yielded multiple engineered variants with increased melting temperatures and improved resistance to thermal unfolding, including FAST-PETase and thermostable cutinases such as LCC-ICCG. These advances demonstrate that enzyme thermostability is, in principle, tunable and have significantly expanded the operational temperature window of PET hydrolases, representing an essential step toward industrial implementation.

**Figure 1:**
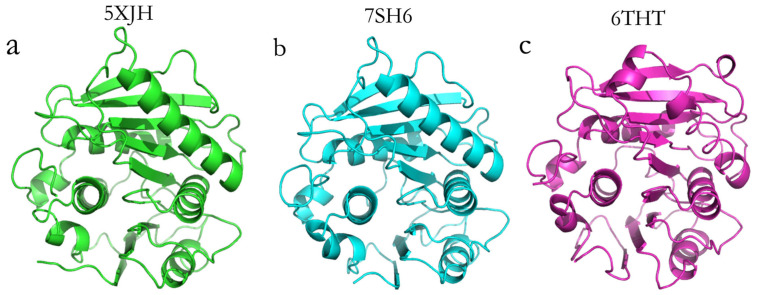
Structural comparison of PETase variants. Cartoon representations of the three PETases: (**a**) WT-PETase (PDB ID: 5XJH); (**b**) FAST-PETase (PDB ID: 7SH6); and (**c**) LCC-ICCG (PDB ID: 6THT). The catalytic triad and major structural elements are labeled.

Despite these successes, enhanced thermal stability alone does not guarantee high-temperature functionality. Several engineered PETases exhibit maximal catalytic activity at temperatures well below PET’s glass transition range, while further heating—although not inducing global unfolding—leads to diminished activity and inconsistent functional outcomes. This behavior reveals a fundamental paradox: enzymes can remain structurally intact yet lose catalytic competence under thermal stress [24]. Notably, this functional divergence arises even though wild-type PETase, FAST-PETase, and LCC-ICCG share highly similar α/β-hydrolase core architectures, with no overt structural rearrangements apparent from static crystal structures (Figure 1). All three PETases share the conserved α/β-hydrolase core fold, while exhibiting distinct structural features in several loop regions and surface elements. These structural differences may contribute to their different thermal adaptations and stability, which are further investigated through molecular dynamics simulations. These observations indicate that thermal deactivation does not necessarily coincide with loss of the native fold, but may instead originate from more subtle disruptions of conformational dynamics and coordinated motions essential for catalysis.

Such findings expose a fundamental limitation of structure-centric interpretations. Elevated temperature can reshape conformational ensembles, alter the population and interconversion of functional substates, and disrupt long-range couplings that coordinate distant structural elements, even when the overall fold remains intact [25]. These effects are inherently dynamic and cannot be inferred from static structures or single-temperature measurements. Consequently, key questions remain unresolved: how is conformational flexibility redistributed across the protein scaffold under thermal stress, which collective motions required for catalysis are preserved or compromised, and how do long-range communication patterns respond to sustained heating? Without addressing these issues in an integrated manner, it is impossible to explain why some PETases retain productive function near PET’s glass transition temperature whereas others become functionally compromised despite comparable apparent stability.

To resolve this missing mechanistic layer, we adopt a comparative temperature-gradient framework to interrogate three representative PET-degrading enzymes—wild-type PETase, FAST-PETase, and LCC-ICCG—spanning distinct thermal adaptation regimes [26,27]. By combining long-timescale molecular dynamics simulations with rigidity analysis and trajectory-derived residue–residue interaction networks, we directly characterize how temperature reorganizes conformational dynamics and long-range coordination. We hypothesized that the enhanced thermostability of engineered PETases results from a more favorable balance between structural rigidity and conformational flexibility, which preserves long-range residue communication and catalytically relevant conformational states under thermal stress. To test this hypothesis, we performed comparative analyses of structural stability, residue flexibility, free energy landscapes, rigidity networks, and residue interaction networks. This integrated approach provides a mechanistic basis for understanding—and ultimately guiding the rational design of—thermally competent PET-degrading enzymes.

## 2. Results

### 2.1. Temperature Stress Reveals Distinct Dynamic Regimes Across PETase Variants

To evaluate the global structural stability of the three PET-degrading enzymes, backbone root-mean-square deviation (RMSD) relative to the initial minimized structures was calculated at each temperature (Supplementary Figure S1).

At 30 °C (Supplementary Figure S1a), all three PET-degrading enzymes maintained stable global conformations throughout the 1 μs molecular dynamics simulations, with no evidence of large-scale unfolding or structural collapse. The backbone RMSD of WT-PETase (5XJH) gradually increased during the first 200 ns and then reached a plateau, fluctuating around 0.20–0.25 nm, indicating an initial structural relaxation followed by equilibration. In contrast, FAST-PETase (7SH6) and LCC-ICCG (6THT) exhibited consistently lower RMSD values over the entire simulation, remaining within a narrow range of approximately 0.05–0.15 nm with minimal fluctuations. These results suggest that although all three enzymes are structurally stable under mild temperature conditions, WT-PETase displays relatively higher conformational flexibility, whereas FAST-PETase and LCC-ICCG possess enhanced backbone rigidity and overall structural stability, likely reflecting the stabilizing effects of protein engineering.

At 50 °C (Supplementary Figure S1b), the overall RMSD trends of the three PET-degrading enzymes represent a clear extension of the behaviors observed at 30 °C, with thermal stress amplifying differences in conformational stability. WT-PETase shows a pronounced increase in RMSD fluctuations compared to 303 K, with the average RMSD level shifting upward and frequent large-amplitude deviations appearing throughout the trajectory, indicating reduced structural robustness and enhanced global flexibility under elevated temperature. FAST-PETase maintains a relatively stable backbone conformation, although moderate RMSD increases and intermittent local fluctuations are observed, suggesting partial thermal perturbation without loss of overall structural integrity. In contrast, LCC-ICCG remains the most stable system at 50 °C, exhibiting the lowest RMSD values and the narrowest fluctuation range among the three enzymes. Collectively, the RMSD profiles reveal a clear stability hierarchy of WT-PETase < FAST-PETase < LCC-ICCG under moderate thermal conditions, highlighting the superior thermostability of LCC-ICCG and the effective, albeit less pronounced, stabilization achieved in FAST-PETase through protein engineering.

At 70 °C (Figure S1c), the RMSD profiles reveal pronounced temperature-induced destabilization effects, with clear distinctions among the three PET-degrading enzymes. WT-PETase exhibits the highest RMSD values and the largest fluctuation amplitudes, frequently oscillating between 0.2 and 0.5 nm, indicative of severe global conformational instability and extensive structural rearrangements under high-temperature conditions. These large deviations, together with elevated RMSF values, suggest that WT-PETase is unable to maintain a stable conformational state that is favorable for catalysis at 70 °C, likely adopting partially unfolded or inactive states. In contrast, FAST-PETase maintains the lowest RMSD levels, predominantly within the 0.1–0.25 nm range, with relatively small fluctuations and no persistent upward drift over time, reflecting superior backbone stability and effective resistance to thermal perturbation. LCC-ICCG displays intermediate behavior, with RMSD values slightly higher than those of FAST-PETase (approximately 0.15–0.3 nm) and the presence of several sharp transient peaks, indicating occasional local conformational disturbances while preserving overall structural integrity. Taken together, the RMSD analysis at 70 °C establishes a clear thermostability and functional resilience ranking of WT-PETase < LCC-ICCG < FAST-PETase, highlighting FAST-PETase as the most structurally stable and catalytically viable enzyme under high-temperature conditions. To assess the reproducibility of the simulation results, an additional independent 1 μs MD simulation was performed for each enzyme at each temperature. The RMSD profiles from the replicate simulations are shown in Supplementary Figure S3. The replicate trajectories reproduced the major temperature-dependent trends observed in the original simulations, including increased conformational fluctuations of WT-PETase at elevated temperatures and the relatively higher structural stability of FAST-PETase and LCC-ICCG. These consistent RMSD patterns confirm the robustness and reproducibility of the observed conformational stability differences among the three PETases.

**Figure 2:**
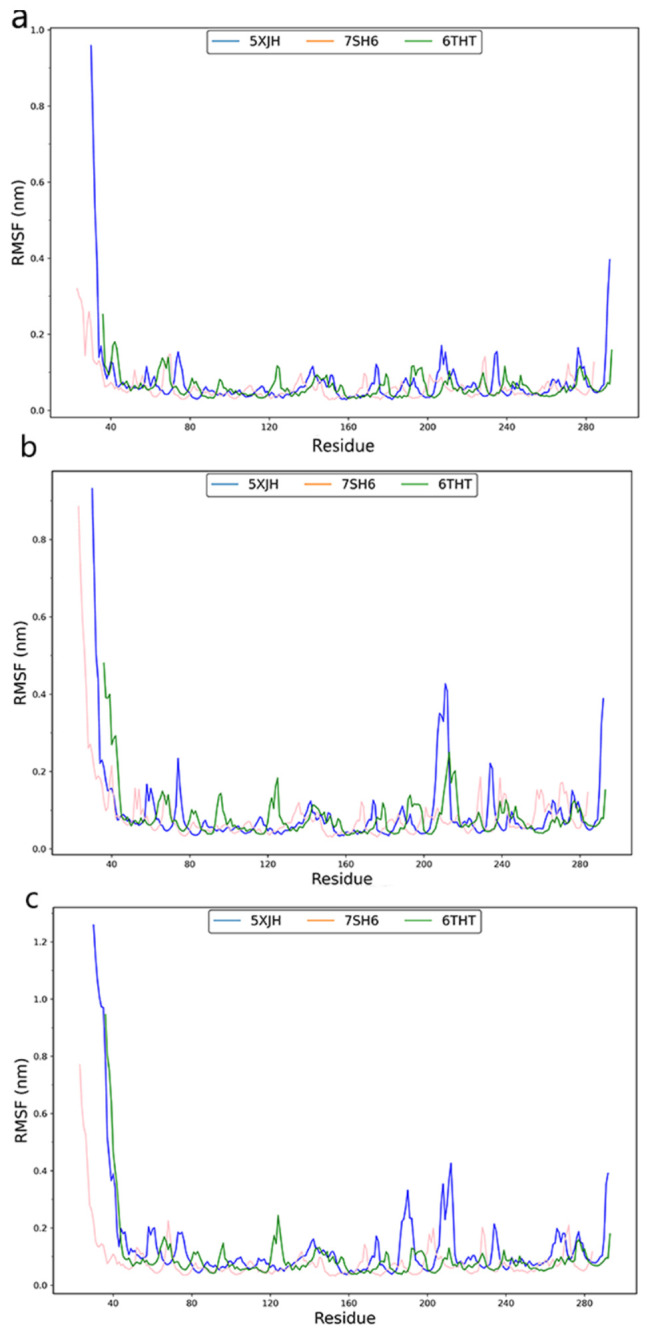
Residue root-mean-square fluctuation (RMSF) profiles of the plastic-degrading enzymes at different temperatures. (**a**) RMSF profiles of the enzymes at 30 °C. (**b**) RMSF profiles of the enzymes at 50 °C. (**c**) RMSF profiles of the enzymes at 70 °C. RMSF values were calculated based on Cα atoms and plotted as a function of residue number. Different curves represent the enzyme structures derived from distinct simulation systems: 5XJH (blue), 7SH6 (orange), and 6THT (green). The RMSF analysis highlights temperature-dependent changes in residue flexibility, particularly in functionally important regions such as the active-site cleft, substrate-binding region, and loop regions involved in PET recognition and catalysis.

To further characterize residue-level flexibility, root-mean-square fluctuation (RMSF) of Cα atoms was computed for each system (Figure 2). While the overall RMSF profiles were broadly comparable, pronounced local differences were observed in loop-dominated regions. In particular, WT-PETase showed elevated fluctuations in several surface-exposed and active-site-adjacent loops, whereas FAST-PETase and LCC-ICCG displayed more restrained flexibility in these regions, especially at higher temperatures. These functionally relevant regions are closely associated with substrate recognition and catalysis, suggesting that modulation of local flexibility plays an important role in the enhanced thermal performance of the engineered and thermophilic enzymes.

At 30 °C, residue-level flexibility was evaluated by calculating the RMSF of Cα atoms for WT-PETase, FAST-PETase, and LCC-ICCG (Figure 2a). Overall, WT-PETase exhibits consistently higher RMSF values across the sequence compared with the two engineered enzymes, indicating increased backbone flexibility. Notably, pronounced fluctuations are observed in the region spanning approximately residues 200–240, suggesting enhanced local mobility in WT-PETase. In contrast, both FAST-PETase and LCC-ICCG maintain uniformly low RMSF values throughout the simulation, with only minor localized fluctuations, reflecting a more rigid and stable structural framework. These results indicate that, even at moderate temperature, WT-PETase displays higher intrinsic flexibility, whereas FAST-PETase and LCC-ICCG exhibit reduced residue-level motion, consistent with their improved conformational stability.

At 50 °C, residue-level flexibility differences among WT-PETase, FAST-PETase, and LCC-ICCG become more pronounced (Figure 2b). WT-PETase exhibits distinct RMSF peaks in the regions spanning residues 60–80 and 200–240, indicating enhanced local mobility upon temperature elevation. These regions correspond primarily to α-helical segments and loop regions, suggesting temperature-induced destabilization of secondary-structure elements in the wild-type enzyme. In contrast, FAST-PETase and LCC-ICCG display highly similar RMSF profiles, with most residues exhibiting fluctuation amplitudes below 0.2 nm and only minor localized variations. The reduced and comparable flexibility observed for FAST-PETase and LCC-ICCG indicates that both enzymes effectively suppress temperature-driven backbone motions, maintaining structural integrity under elevated thermal conditions.

At 70 °C, pronounced differences in residue-level flexibility are observed among the three PET-degrading enzymes (Figure 2c). WT-PETase exhibits markedly elevated RMSF values at both the N- and C-termini, as well as in the central region spanning residues 180–220, indicating severe destabilization under high-temperature conditions. These regions largely correspond to loop and peripheral structural elements, suggesting extensive thermally induced backbone fluctuations and partial loss of structural integrity. In contrast, FAST-PETase displays the lowest RMSF values across the entire sequence, with most residues fluctuating below 0.2 nm, reflecting a highly rigid and thermally robust backbone. LCC-ICCG also maintains generally low RMSF values; however, distinct local peaks are observed in specific regions, notably around residues 110–130 and near residue 200. This behavior suggests that while LCC-ICCG preserves a stable global fold comparable to FAST-PETase, it retains enhanced flexibility in selected loop regions. Such a “stable scaffold with flexible functional loops” dynamic mode may facilitate substrate accommodation and catalytic activity at elevated temperatures. To further examine whether the residue-level flexibility characteristics were preserved across independent trajectories, we performed an additional 1 μs MD simulation for each enzyme at each temperature (Supplementary Figure S4). The replicate RMSF profiles showed highly consistent distributions of flexible and stable regions compared with those observed in the original simulations. In particular, the increased fluctuations of WT-PETase in loop-rich regions and the restrained flexibility of FAST-PETase and LCC-ICCG were consistently reproduced, supporting the reliability of the temperature-dependent residue dynamics identified in this study.

### 2.2. Temperature Reshapes Conformational Ensembles and Destabilizes Functional Basins

**Figure 3:**
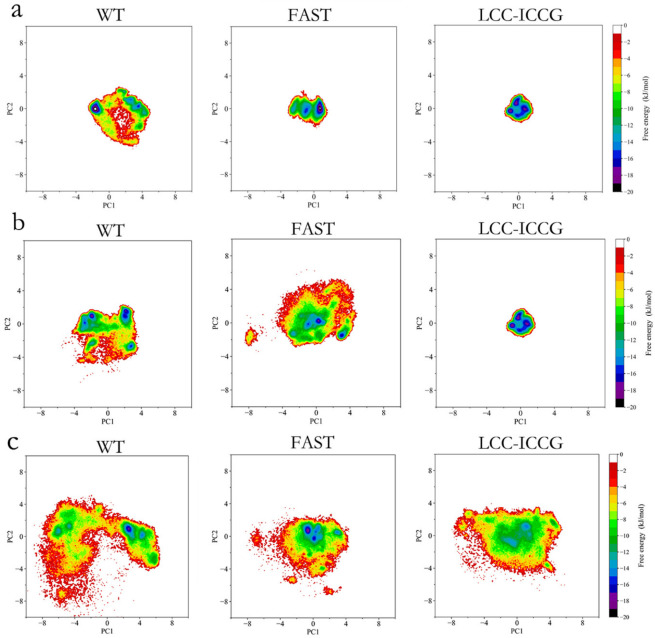
Temperature-dependent free energy landscapes (FELs) of PET-degrading enzymes derived from principal component analysis (PCA). The FELs were constructed by projecting molecular dynamics trajectories onto the first two principal components (PC1 and PC2). Panels (**a**–**c**) correspond to simulations performed at 30 °C, 50 °C, and 70 °C, respectively. In each row, the columns represent WT-PETase (**left**), FAST-PETase (**middle**), and LCC-ICCG (**right**). The color-coded contours indicate the relative free energy (kJ/mol), where blue regions represent low-energy basins corresponding to more stable conformational states, and red regions indicate higher-energy conformations.

While RMSF analysis revealed residue-level flexibility differences, it cannot fully explain how local fluctuations influence global conformational stability. Therefore, to characterize the thermodynamic behavior of WT-PETase, FAST-PETase, and LCC-ICCG, two-dimensional free-energy landscapes (FELs) were constructed by projecting the MD trajectories onto the first two principal components (PC1 and PC2) across three temperatures (30, 50, and 70 °C). At 30 °C (Figure 3a), WT-PETase exhibited a relatively broad and dispersed free-energy distribution with multiple shallow minima, indicating pronounced conformational heterogeneity and several metastable states. In contrast, FAST-PETase showed a more compact basin centered around a dominant low-energy minimum, reflecting reduced conformational diversity and improved global stability. LCC-ICCG displayed the most localized and deepest free-energy minimum, with conformational sampling confined to a narrow region of PC space, suggesting a highly stabilized structural ensemble. Upon increasing the temperature to 50 °C (Figure 3b), the free-energy landscape of WT-PETase became substantially broadened and fragmented, indicating reduced thermodynamic stability, whereas FAST-PETase retained a relatively compact basin with moderate expansion of conformational sampling, suggesting preserved stability accompanied by adaptive flexibility. LCC-ICCG remained tightly confined with a well-defined minimum, demonstrating strong resistance to thermal perturbation. At 70 °C (Figure 3c), these differences became more pronounced: WT-PETase displayed a highly dispersed and rugged landscape with the loss of a dominant energy basin, implying severe conformational destabilization; FAST-PETase maintained a partially organized free-energy profile with broadened substates, indicating intermediate thermal tolerance; and LCC-ICCG still exhibited a compact and deep minimum, reflecting a strongly stabilized conformational ensemble even under extreme thermal stress. Collectively, the temperature-dependent divergence of the FELs establishes a clear stability hierarchy of LCC-ICCG > FAST-PETase > WT-PETase and links the suppression of local flexibility observed in RMSF to progressively restricted and thermodynamically favorable conformational sampling. We analyzed the free-energy landscapes derived from the independent replicate simulations to evaluate the consistency of the observed thermodynamic behaviors (Supplementary Figure S5). The replicate trajectories reproduced the major energy basins and overall conformational distributions observed in the original simulations across all three temperatures. The preservation of compact low-energy regions in FAST-PETase and LCC-ICCG, together with the broader and more dispersed energy distributions of WT-PETase at elevated temperatures, indicates that the distinct thermodynamic characteristics identified in the original simulations are not dependent on a single trajectory. These results support the robustness of the temperature-dependent conformational landscapes.

### 2.3. Mechanical Scaffold Coherence Underlies Ensemble Stability at High Temperature

**Figure 4:**
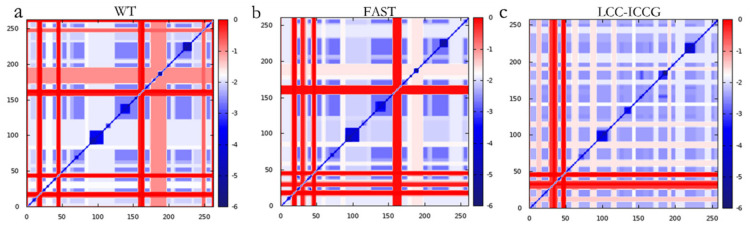
CNAnalysis stability maps of PET-degrading enzymes. Stability maps of (**a**) WT-PETase, (**b**) FAST-PETase, and (**c**) LCC-ICCG resulting from thermal unfolding simulations of a single constraint network derived from the corresponding crystal structures (PDB accessions 5XJH, 7SH6, and 6THT, respectively). The red color indicates pairs of residues forming weak rigid contacts, whereas increasing blue intensity corresponds to progressively stronger rigid contacts. These maps provide a residue-level representation of rigidity distribution and long-range constraint coupling within each enzyme.

To further elucidate the structural origin underlying the distinct free energy landscapes observed among the three plastic-degrading enzymes, rigidity analysis was performed to characterize their residue–residue mechanical coupling patterns. As shown in Figure 4a, WT-PETase exhibits a highly fragmented rigidity network, with discontinuous and irregularly distributed rigid clusters interspersed with extensive flexible regions. Although several rigid segments persist, their weak interconnection indicates a loss of global mechanical coherence, providing a structural explanation for the dispersed and rugged free energy landscape observed at elevated temperature. In contrast, FAST-PETase displays a more organized rigidity pattern (Figure 4b), characterized by several dominant rigid bands spanning across the matrix, suggesting the presence of a partially preserved mechanical scaffold that stabilizes the protein while still allowing moderate conformational adaptability. Notably, LCC-ICCG presents a highly ordered and dense rigidity network, with extensive long-range mechanical couplings connecting multiple structural regions (Figure 4c). This well-integrated rigidity architecture effectively constrains large-scale conformational fluctuations, consistent with its compact and well-defined free energy minimum. Together, the rigidity analysis reveals a clear hierarchy in mechanical stability among the three enzymes, providing a mechanistic basis for the temperature-dependent differences observed in their free energy landscapes.

### 2.4. Coupling Persistence and Pathway Robustness Define High-Temperature Functional Resilience

To further characterize the spatial organization of protein dynamics and enable domain-level interaction analysis, the RMSF profiles of each enzyme were systematically examined across all simulated temperatures. Based on the temperature-dependent RMSF profiles shown in Figure S2, each enzyme was further partitioned into six dynamic domains for subsequent NRI analysis. The domain boundaries were determined according to the magnitude and continuity of residue-level fluctuations, such that residues exhibiting similar mobility patterns were grouped into the same region. This RMSF-guided partitioning allowed flexible loop segments, moderately dynamic secondary-structure elements, and relatively rigid core regions to be distinguished in a physically meaningful manner. Owing to differences in sequence length, structural composition, and thermal response among WT-PETase (Figure S2a), FAST-PETase (Figure S2b), and LCC-ICCG (Figure S2c), the exact domain boundaries varied slightly between proteins. Nevertheless, in all cases, the six-domain division consistently captured major fluctuation hotspots and structurally coherent regions, providing a unified yet protein-specific framework for constructing domain-level interaction networks in the NRI model.

**Figure 5:**
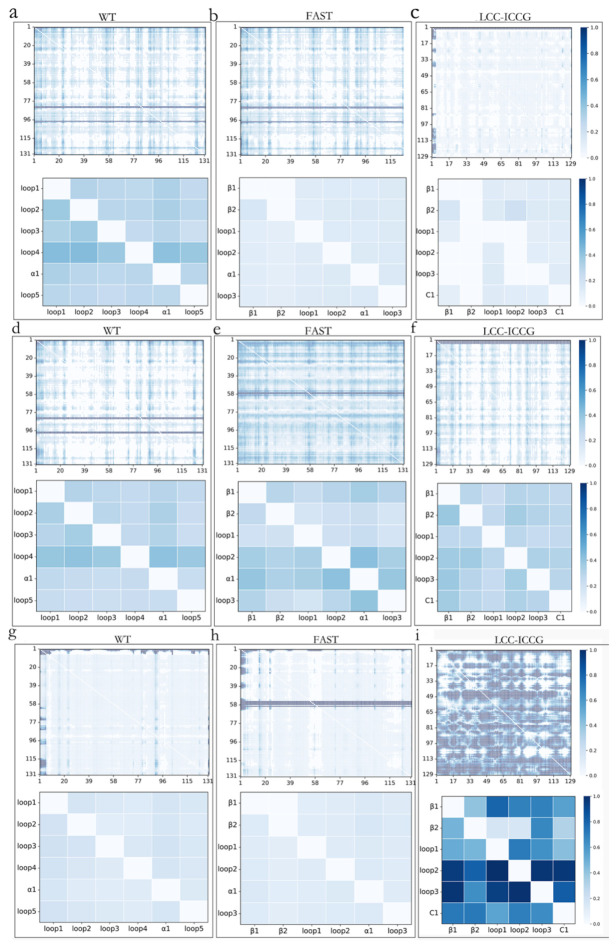
Temperature-dependent residue–residue and domain-level interaction networks inferred by Neural Relational Inference (NRI). Panels (**a**–**c**), (**d**–**f**), and (**g**–**i**) correspond to simulations at 30 °C, 50 °C, and 70 °C, respectively, with WT-PETase, FAST-PETase, and LCC-ICCG shown from left to right within each temperature group. For each panel, the upper heatmap represents the residue–residue dynamic cross-correlation matrix (DCCM) derived from MD trajectories, where color intensity indicates the magnitude of correlated motions. The lower matrix shows the domain-averaged correlation coefficients, highlighting collective coupling between structural regions. Increasing temperature leads to progressive weakening and redistribution of correlated motions in WT-PETase, moderate reorganization in FAST-PETase, and comparatively preserved or enhanced domain-level coupling in LCC-ICCG, consistent with their distinct thermal stability profiles.

Based on the RMSF-guided domain partitioning, NRI analysis was employed to compare residue–residue interaction networks of the three PET-degrading enzymes at 303 K (Figure 5a–c). WT-PETase exhibits the strongest and most extensive interaction network at this temperature, with high interaction intensities spanning multiple loop regions and structural domains. The pronounced inter-domain coupling suggests efficient mechanical communication and a well-balanced interplay between flexibility and stability, consistent with WT-PETase being optimally adapted to moderate temperatures.

In comparison, FAST-PETase displays an overall reduction in interaction strength at 303 K, with weakened coupling particularly involving flexible loop regions, indicating that its allosteric communication network is not fully activated at lower temperatures. LCC-ICCG shows a further attenuation of residue- and domain-level interactions, reflecting a largely disengaged communication architecture at 303 K. Together, these observations suggest that as the intrinsic temperature optimum of the enzyme increases from WT to FAST to LCC, the interaction network becomes progressively less active at low temperature, highlighting distinct temperature-dependent allosteric adaptation mechanisms among the three enzymes.

To further elucidate the temperature-dependent functional divergence among PETases, we compared the NRI interaction patterns of WT-PETase, FAST-PETase, and LCC-ICCG at 323 K (Figure 5d–f). At this temperature, which exceeds the optimal operating range of WT-PETase, a pronounced weakening of the interaction network is observed. In the residue–residue heatmap, WT-PETase exhibits diffuse and fragmented interaction patterns, with a marked loss of continuous high-intensity regions. Correspondingly, the domain–domain interaction matrix reveals substantially reduced inter-domain coupling, particularly among loop regions and the α-helical core, indicating a breakdown of long-range cooperative motions. This widespread attenuation of residue- and domain-level interactions suggests that WT-PETase undergoes functional deactivation at 323 K, as excessive thermal fluctuations disrupt the mechanical communication required for coordinated catalytic dynamics.

In contrast, FAST-PETase, which is thermally adapted to intermediate temperatures, maintains a relatively robust interaction network at 323 K. Both the residue-level and domain-level NRI maps display enhanced and more uniformly distributed interaction strengths compared to WT-PETase, reflecting preserved inter-domain coupling and efficient dynamic communication. Notably, interactions involving flexible loop regions remain strongly connected to the structural core, indicating an optimal balance between conformational flexibility and global coordination that supports conformational states associated with catalysis at this temperature.

Strikingly, LCC-ICCG exhibits the most compact and stable interaction network at 323 K. Its residue–residue heatmap is dominated by highly localized and intense interaction clusters, while the domain–domain matrix shows consistently strong intra- and inter-domain coupling. This highly cohesive interaction pattern reflects exceptional thermal robustness, enabling LCC-ICCG to maintain structural integrity and functional dynamics even under elevated thermal conditions. Collectively, these results demonstrate a clear hierarchy of thermal adaptability at 323 K, with WT-PETase undergoing interaction network collapse and loss of coordinated conformational dynamics, FAST-PETase operating near its optimal dynamic regime, and LCC-ICCG retaining superior thermostability through tightly coordinated residue- and domain-level interactions.

Upon further increasing the temperature to 343 K (Figure 5g–i), the contrast in interaction network robustness among the three PETases becomes even more pronounced. At this high-temperature condition, WT-PETase exhibits a near-complete collapse of its residue–residue interaction network, as evidenced by the largely diffuse and low-intensity NRI heatmap. Consistently (Figure 5g), the corresponding domain–domain interaction matrix shows uniformly weak coupling across all structural regions, indicating a severe disruption of long-range cooperative motions. These results suggest that WT-PETase loses effective mechanical communication at 343 K, rendering it dynamically disordered and functionally inactive.

FAST-PETase displays an intermediate response at 343 K (Figure 5h). Although residual interaction patterns remain detectable at both the residue and domain levels, the overall interaction strength is markedly reduced compared with its behavior at moderate temperatures. The weakening of inter-domain coupling, particularly between loop regions and the α-helical core, implies that FAST-PETase approaches the upper limit of its thermal tolerance, with diminished dynamic coordination under extreme thermal stress.

In striking contrast, LCC-ICCG maintains a dense and highly coherent interaction network at 343 K (Figure 5i). Its residue–residue NRI heatmap is characterized by extensive high-intensity interaction clusters distributed across the protein, while the domain–domain matrix reveals exceptionally strong and persistent coupling among β-strands, loop regions, and the catalytic core (C1). Notably, loop2 and loop3 exhibit pronounced interactions with multiple structural domains, forming a tightly integrated network that supports efficient long-range communication even at elevated temperatures. This robust interaction architecture highlights the superior thermal adaptability of LCC-ICCG, suggesting that its high-temperature activity is enabled by a globally reinforced yet dynamically connected interaction network. Together, these findings demonstrate that LCC-ICCG achieves genuine thermostability at 343 K, in sharp contrast to the interaction network collapse observed for mesophilic and intermediate-temperature PETases.

**Figure 6:**
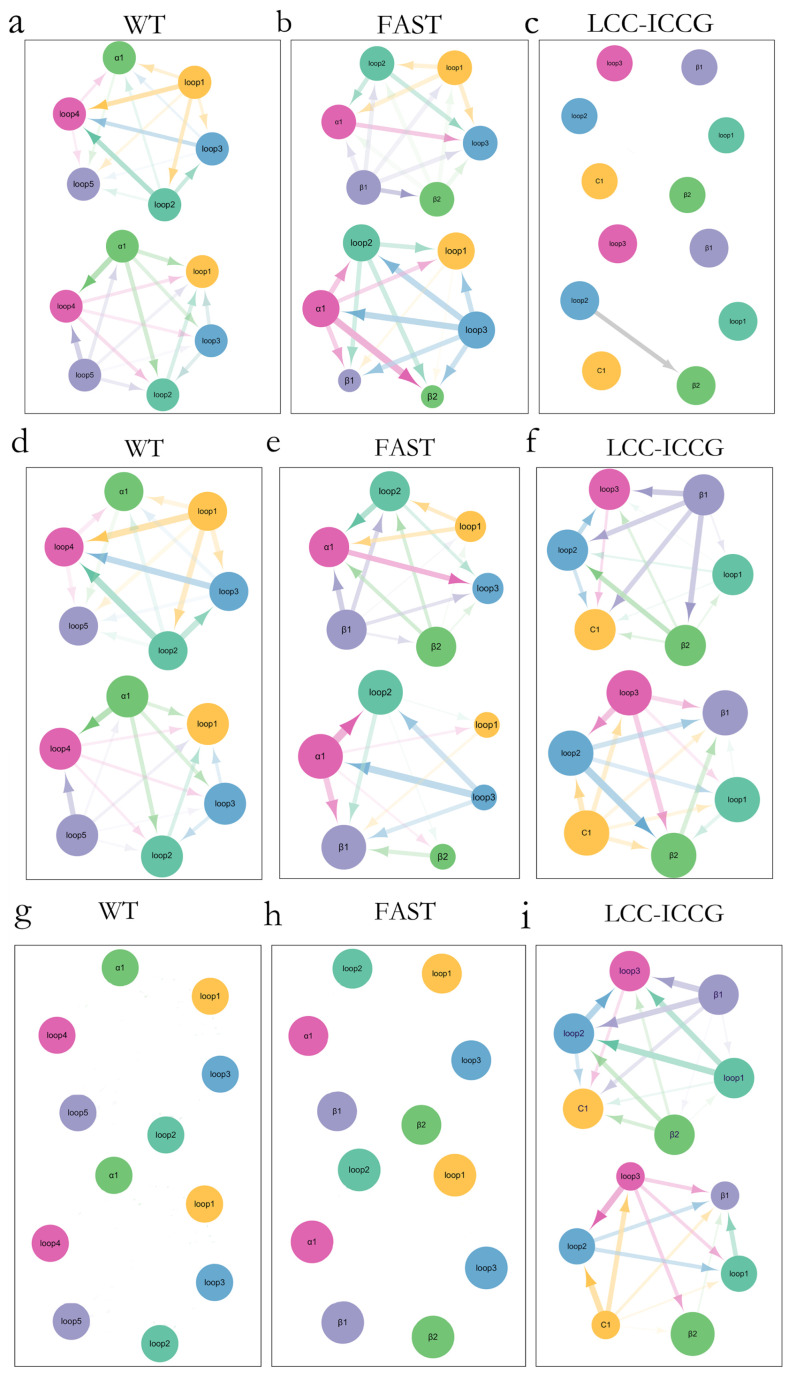
Temperature-dependent inter-domain signaling and residue-level communication networks of PET-degrading enzymes inferred by NRI analysis. Panels (**a**–**c**), (**d**–**f**), and (**g**–**i**) correspond to 30 °C, 50 °C, and 70 °C, respectively. Within each temperature group, WT-PETase, FAST-PETase, and LCC-ICCG are shown from left to right. In each panel, the diagram represents domain-level signaling interactions. Nodes indicate structural domains, and edges represent dynamic coupling. Increasing temperature leads to progressive network fragmentation in WT-PETase, partial reorganization in FAST-PETase, and relatively preserved connectivity in LCC-ICCG, highlighting enhanced thermal robustness in engineered variants.

To further characterize how residue- and domain-level interactions are organized into effective information transfer pathways, NRI-based communication networks were constructed at 303 K (Figure 6). The domain-level directed interaction pathways shown in the upper panels were visualized using *Cytoscape*, while the lower panels represent the corresponding residue-level transmission networks.

For WT-PETase, the pathway analysis reveals a dense and highly integrated communication architecture at 303 K. Multiple loop regions (loop1–loop4) function as central hubs, actively mediating bidirectional information transfer between the α1 helix and surrounding structural elements. In particular, loop2 and loop3 form dominant conduits that couple flexible surface regions to the catalytic core, consistent with their RMSF-identified dynamical significance. At the residue level, WT-PETase exhibits a broadly distributed and highly connected transmission network, indicating efficient long-range communication across the protein. This hierarchical organization—from extensive residue-level pathways to coherent domain-level routes—suggests that WT-PETase achieves optimal allosteric communication and mechanical coupling at its native working temperature, thereby supporting frequent conformational transitions required for catalytic activity.

FAST-PETase displays a more streamlined yet highly directional communication topology at 303 K. Compared with WT, information flow is concentrated into fewer dominant pathways, primarily involving β-strands (β1–β2), loop regions, and the α1 helix. This redistribution results in a more focused and efficient transmission network with reduced redundancy, reflecting an engineered communication strategy that balances flexibility and robustness and contributes to its enhanced performance across a broader temperature range.

In contrast, LCC-ICCG exhibits a markedly simplified communication network at 303 K. Both the Cytoscape-based domain pathways and the residue-level transmission graph show limited active routes, with information flow largely confined to localized loop–core interactions. The absence of extensive long-range pathways indicates that LCC-ICCG adopts a restrained dynamical state at moderate temperatures, suppressing collective motions and inter-domain coupling. This behavior suggests that LCC-ICCG does not fully activate its communication network at 303 K but instead preserves interaction capacity for high-temperature conditions, consistent with its thermophilic adaptation.

Upon increasing the temperature to 323 K, pronounced differences emerge in the NRI-derived communication pathways among WT-, FAST-, and LCC-type PETases (Figure 6d–f). For WT-PETase, the domain-level pathway network becomes markedly fragmented compared with its well-integrated topology at 303 K. Several previously dominant communication routes weaken or disappear, and information transfer is no longer efficiently propagated between loop regions and the α1 helix. This loss of coherent long-range pathways is further reflected at the residue level, where the transmission network appears sparse and discontinuous, indicating a breakdown of collective motions and effective allosteric coupling. These features suggest that WT-PETase undergoes functional deactivation at 323 K, consistent with its limited thermal tolerance and the elevated structural fluctuations observed in RMSF and free-energy analyses.

In contrast, FAST-PETase exhibits a highly organized and directional communication architecture at 323 K. The Cytoscape-based domain pathway network reveals strong and persistent information flow among β-strands, loop regions, and the α1 helix, forming a compact yet efficient transmission core. Compared with WT, the number of effective pathways is reduced but strategically concentrated, minimizing redundant communication while preserving strong inter-domain coupling. At the residue level, FAST-PETase displays a well-connected network with clearly defined high-importance nodes, indicating robust long-range communication and coordinated dynamics. This optimized pathway organization highlights FAST-PETase’s adaptation to intermediate temperatures and explains its sustained catalytic competence at 50 °C.

LCC-ICCG shows a distinct communication strategy at 323 K, characterized by relatively weak and localized interaction pathways. Both the domain-level and residue-level networks display limited long-range information transfer, with interactions primarily confined within local structural modules. Notably, β-sheet regions and the C1 domain maintain internal connectivity, while cross-domain coupling remains suppressed. This restrained communication pattern suggests that LCC-ICCG does not fully activate its allosteric network at 323 K but instead preserves structural integrity and interaction potential for higher-temperature conditions. Such behavior is consistent with its thermophilic nature, whereby global communication pathways are selectively enhanced only under high-temperature environments.

At 343 K, the NRI-derived signal propagation networks exhibit a striking divergence among WT-, FAST-, and LCC-type PETases, reflecting their fundamentally different thermal adaptation strategies (Figure 6g–i). Notably, the interaction network of WT-PETase is essentially absent at this temperature, with no coherent domain-level or residue-level communication pathways detected. This complete collapse of signal transmission indicates a loss of effective allosteric coupling and collective motion, consistent with severe structural destabilization and loss of coordinated conformational dynamics of WT-PETase under high-temperature conditions.

FAST-PETase shows a highly reduced communication network at 343 K, characterized by the persistence of only a single dominant transmission pathway. Both the Cytoscape-based domain network and the residue-level pathway map reveal that information flow becomes strongly constrained and localized, with most long-range connections eliminated. This simplified topology suggests that FAST-PETase is approaching the upper limit of its thermal tolerance: while partial communication is still maintained, the global allosteric network required for efficient catalysis is no longer fully supported.

In contrast, LCC-ICCG retains a well-defined and highly connected signal propagation network at 343 K. The domain-level pathways reveal strong and multidirectional information flow among β-strands, loop regions, and the C1 domain, forming a dense and integrated communication architecture. At the residue level, multiple high-importance nodes and continuous transmission routes are preserved, indicating robust long-range coupling and coordinated dynamics even at elevated temperatures. Unlike WT and FAST, LCC-ICCG does not exhibit network fragmentation; instead, its allosteric communication remains globally organized, supporting efficient redistribution of thermal fluctuations across the protein. This sustained and structured signal transmission network provides a mechanistic explanation for the exceptional thermostability and catalytic competence of LCC-ICCG at 70 °C, highlighting its ability to maintain functional dynamics through reinforced inter-domain coupling under extreme thermal stress.

Taken together, the temperature-dependent analyses reveal a progressive reorganization of structural dynamics and allosteric communication across the three PETase variants. As temperature increases, WT-PETase undergoes a continuous loss of conformational control, manifested by elevated flexibility, dispersed free-energy landscapes, weakened residue–residue interactions, and ultimately a complete collapse of signal propagation networks at 343 K. FAST-PETase displays an intermediate response, maintaining partial structural integrity and simplified communication pathways up to 323 K, but exhibiting pronounced network reduction and constrained information flow at 343 K, indicative of an approaching thermal limit. In contrast, LCC-ICCG preserves a coherent and highly connected interaction network even at 343 K, characterized by stable free-energy basins, restrained global flexibility, and robust inter-domain signal transmission. These results collectively demonstrate that the three enzymes adopt distinct temperature-adaptive dynamic regimes, with thermal tolerance closely associated with the preservation of global allosteric communication rather than mere local rigidity.

**Figure 7:**
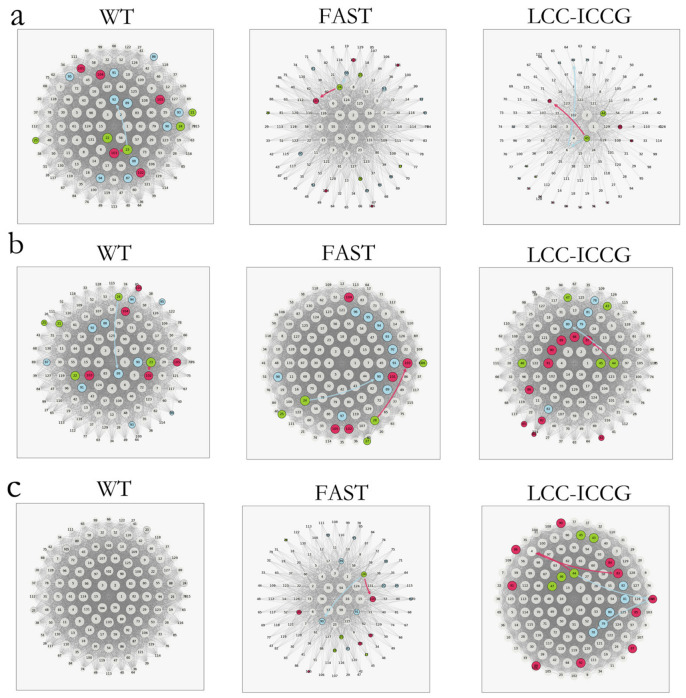
Comparison of residue-level communication networks derived from Neural Relational Inference (NRI) for WT-PETase, FAST-PETase, and LCC-ICCG at different temperatures. Panels (**a**–**c**) correspond to 303 K, 323 K, and 343 K, respectively. Colored nodes indicate residues with high communication importance, whereas gray nodes represent residues with relatively low network centrality. Edges represent dominant information transfer pathways inferred from the NRI model. The figure summarizes the progressive reorganization of residue communication networks with increasing temperature, highlighting the distinct thermal adaptation strategies of the three PETases.

To facilitate comparison of the temperature-dependent communication patterns among the three PETases, the residue-level signal propagation networks at 303, 323, and 343 K are summarized in Figure 7. The graphical comparison clearly illustrates the progressive simplification of the interaction network in WT-PETase, the intermediate communication architecture retained by FAST-PETase, and the highly connected network preserved in LCC-ICCG under elevated temperatures. These visualized network topologies provide an intuitive overview of the distinct thermal adaptation strategies inferred from the NRI analyses and complement the detailed pathway descriptions presented above.

## 3. Discussion

By integrating molecular dynamics simulations with neural relational inference, our analyses reveal that WT-PETase, FAST-PETase, and LCC-ICCG adopt distinct dynamic strategies for thermal adaptation, reflecting different evolutionary and engineering constraints.

WT-PETase exemplifies a flexibility-driven adaptation mechanism optimized for moderate temperatures. Its conformational dynamics are characterized by dynamic loop motions and transient inter-domain coupling that facilitate frequent conformational rearrangements, features that are generally considered important for catalysis. However, this strategy lacks robustness against thermal perturbation. As temperature increases, the same flexibility that favors conformational adaptability at lower temperatures becomes excessive, leading to the disruption of coordinated motions and fragmentation of the allosteric network. This behavior highlights a key limitation of naturally evolved mesophilic enzymes: functional flexibility is achieved at the expense of network resilience, rendering them vulnerable to global communication failure under heat stress.

In contrast, FAST-PETase follows a rigidity-stabilization strategy characteristic of engineered intermediate-temperature enzymes. Rather than relying on extensive conformational sampling, FAST-PETase achieves enhanced thermal adaptation by reinforcing intra-domain packing and suppressing large-scale fluctuations. This structural consolidation preserves coherent signal transmission across a broad temperature range, albeit with reduced conformational diversity. Such a mechanism suggests that FAST-PETase trades dynamic richness for thermal robustness, enabling stable catalytic performance under elevated but not extreme thermal conditions.

LCC-ICCG represents a distinct network-resilient adaptation paradigm. Its high-temperature tolerance cannot be attributed solely to increased rigidity. Instead, LCC-ICCG combines a stable structural framework with strategically retained flexibility in functional regions, supported by a highly interconnected and redundant allosteric network. This distributed communication architecture allows perturbations to be rerouted through alternative pathways, preventing catastrophic loss of signal transmission at high temperature. Consequently, thermal stability in LCC-ICCG emerges as a property of network organization rather than local structural rigidity alone.

Collectively, these findings underscore that enzymatic thermostability arises from qualitatively different adaptive solutions rather than a monotonic increase in rigidity. Flexibility-dominated, rigidity-dominated, and network-resilient strategies represent three distinct modes of thermal adaptation, each with inherent advantages and trade-offs. From a protein engineering perspective, these results suggest that enhancing high-temperature activity requires not only stabilizing secondary structures but also deliberately redesigning allosteric communication networks to maintain functional coherence under thermal stress. This network-centric view of enzyme adaptation provides a conceptual framework for rationally engineering robust biocatalysts capable of operating across diverse temperature regimes.

Previous studies have demonstrated that enzyme thermostability is governed by multiple structural determinants, including enhanced hydrophobic core packing, optimized hydrogen-bonding networks, strengthened electrostatic interactions such as salt bridges, and an appropriate balance between structural rigidity and conformational flexibility. Our findings complement these established principles by demonstrating that these structural determinants are dynamically integrated through long-range residue communication. Rather than acting independently, structural stability and dynamic coordination jointly determine the ability of PETases to preserve conformational states favorable for catalysis under thermal stress. Therefore, the superior thermal adaptation observed in FAST-PETase and LCC-ICCG reflects not only improvements in classical structural stabilization but also the maintenance of robust allosteric communication networks across different temperature regimes.

Despite these mechanistic insights, several limitations of the present study should be acknowledged. First, the conclusions are primarily based on molecular dynamics simulations and computational network analyses and therefore require further validation through complementary experimental approaches, such as mutagenesis, thermostability measurements, and enzymatic activity assays. Second, only three representative PETases were investigated, which may not fully represent the diversity of thermal adaptation mechanisms across the broader PET hydrolase family. Finally, the simulations were performed under predefined conditions and finite timescales, which may not capture all biologically relevant conformational events. Future studies integrating experimental validation, additional PETase variants, and extended simulations will further strengthen and generalize the proposed mechanistic framework.

## 4. Materials and Methods

### 4.1. Protein Model Construction

To investigate the temperature-dependent conformational dynamics of PET-degrading enzymes, we constructed molecular models of three representative hydrolases: wild-type PETase (WT-PETase), the engineered variant FAST-PETase, and the thermostable cutinase mutant LCC-ICCG. Crystal structures with high resolution were selected as modeling templates, including WT-PETase (PDB ID: 5XJH) [28], FAST-PETase (PDB ID: 7SH6) [29], and LCC-ICCG (PDB ID: 6THT) [30]. These structures encompass all key catalytic regions of α/β-hydrolase fold enzymes, including the catalytic triad (Ser–His–Asp), the oxyanion hole, and flexible loops surrounding the substrate-binding cleft. Missing loops, unresolved terminal residues, and incomplete side chains were reconstructed using MODELLER-10.4 [31], ensuring structural completeness prior to simulation. For FAST-PETase and LCC-ICCG, all engineered substitutions (e.g., N233K, R224Q, S121E in FAST-PETase; ICGG mutations in LCC) were maintained as resolved in their respective crystallographic structures, requiring no additional mutagenesis.

All structures were subjected to 10,000 steps of energy minimization using the conjugate gradient algorithm under the AMBER99SB-ILDN [32] force field. This procedure relieved steric clashes, optimized hydrogen-bonding networks, and relaxed local geometries without distorting native fold topology. The minimized models were further evaluated through stereochemical assessments to ensure appropriate backbone dihedral distributions and side-chain rotamer states.

The resulting optimized structures served as initial conformations for subsequent long-timescale molecular dynamics simulations [33] at 30 °C, 50 °C, and 70 °C. These simulations allowed us to systematically probe temperature-driven changes in structural stability, loop flexibility, and domain interactions among WT-PETase, FAST-PETase, and LCC-ICCG. Combined with Constraint Network Analysis (CNAnalysis) [34], Free Energy Surface (FES) calculations, and Neural Relational Inference (NRI) modeling, these structural models provided a validated foundation for investigating the molecular determinants of enzyme thermostability and catalytic adaptation.

### 4.2. Constraint Network Analysis (CNAnalysis)

Thermal unfolding simulations on an ensemble of networks derived from crystal structures of WT-PETase, FAST-PETase and LCC-PETase (PDB accessions 5XJH, 7SH6 and 6THT, respectively) were performed utilizing the CNAnalysis Web Interface (http://www.cnanalysis.de) [35]. Overall, 50 network topologies were used for thermal unfolding simulations. Hydrophobic tethers were selected to stay constant during the simulations. Default E-cutoffs with initial value −0.1, terminal value −6.0 and stepsize 0.1 were selected. The stability map was generated analogously but was derived from a thermal unfolding simulation of a single network.

### 4.3. Molecular Dynamics Simulation

To investigate the temperature-dependent conformational stability and dynamic behavior of PET-degrading enzymes, we performed all-atom molecular dynamics (MD) simulations on wild-type PETase (WT-PETase), FAST-PETase, and the thermostable cutinase variant LCC-ICCG using GROMACS-2023 [36]. All simulations were conducted under the AMBER99SB-ILDN force field, which has been widely validated for protein dynamics studies.

Each system, including wild-type PETase (WT-PETase, PDB ID: 5XJH), LCC-PETase (PDB ID: 6THT), and FAST-PETase (PDB ID: 7SH6), was solvated in a cubic box using explicit TIP3P [37] water molecules. Counterions (Na^+^ and Cl^−^) were added to neutralize the system and maintain a physiological salt concentration of 150 mM. Energy minimization was performed using the steepest descent algorithm to eliminate steric clashes. Equilibration was conducted sequentially under NVT and NPT ensembles, maintaining temperatures of 30 °C (303 K), 50 °C (323 K), and 70 °C (343 K) using a velocity rescaling thermostat (τ = 0.1 ps) and a pressure of 1 atm using the Parrinello–Rahman barostat (τ = 0.5 ps). All bond lengths were constrained using the LINCS algorithm, and a 2 fs integration timestep was used. Long-range electrostatics were calculated with the particle mesh Ewald (PME) [38] method with a real-space cutoff of 1.0 nm. Lennard-Jones interactions were truncated at 1.0 nm, and a long-range dispersion correction was applied to energy and pressure.

Each molecular dynamics (MD) simulation was run for 1 µs to allow extensive sampling of conformational space. By maintaining the system under standard conditions, this protocol enabled the system to explore a range of conformations, including intermediate and activated-like states, which might otherwise require longer simulation times to observe under conventional MD timescales.

### 4.4. Conformational Flexibility and Molecular Motion Analysis

To evaluate the structural stability and dynamic characteristics of different PET-degrading enzymes under varying temperature conditions, this study conducted a systematic analysis of molecular dynamics trajectories, focusing on overall conformational shifts, residue-scale flexible fluctuations, and functionally relevant collective conformational movements.

Backbone root-mean-square deviation (RMSD) was calculated using the ‘gmx rms’ module [39] in GROMACS to quantify the deviation of protein backbone atoms from the initial minimized structure as a function of simulation time. This analysis was employed to evaluate the overall conformational stability of WT-PETase, FAST-PETase, and LCC-ICCG under different temperature conditions throughout the simulations.

In addition, root-mean-square fluctuation (RMSF) values of Cα atoms were computed using the ‘gmx rmsf’ [40] tool to characterize residue-level flexibility. Particular attention was paid to functionally important regions, including the catalytic active-site loops, substrate-binding cleft, and flexible surface loops surrounding the α/β-hydrolase core, which are known to play critical roles in substrate recognition and thermal adaptation.

### 4.5. Neural Relational Inference

To analyze temperature-dependent changes in residue–residue interaction patterns and dynamic communication networks within PET-degrading enzymes, we employed the Neural Relational Inference (NRI) framework—a graph-based deep learning model designed to infer latent interaction graphs from time-series data [41,42]. This approach integrates protein structural dynamics with probabilistic graph inference, enabling the identification of both spatially proximal and long-range dynamic couplings among residues in WT-PETase, FAST-PETase, and LCC-ICCG under different thermal conditions.

Input features were extracted from molecular dynamics trajectories of each PET-degrading enzyme. To reduce computational cost while preserving the dominant dynamical information required for graph inference, the MD trajectories were uniformly downsampled to 5000 frames. Because the computational complexity of the NRI model increases substantially with the number of input nodes, only representative αC atoms were retained for subsequent analysis. The selected residues were uniformly sampled across the entire protein after RMSF-guided domain partitioning, ensuring that all six dynamic domains and the major flexible regions were represented in the NRI model rather than restricting the analysis to a continuous segment of the sequence. This procedure yielded 131 residues for WT-PETase, 131 residues for FAST-PETase, and 129 residues for LCC-ICCG. Because all major fluctuation hotspots and domain-level communication regions were preserved, the downsampling strategy was designed to reduce computational cost while maintaining the overall topology of the residues. For each frame, 3D Cartesian coordinates and velocity vectors were used, yielding an (N × 6) feature matrix, where N denotes the number of selected residues. All features were min–max normalized to the range [−1, 1]. Temporal sequences were generated using a sliding window of 50 frames with a stride of 100 frames to capture time-dependent residue–residue interactions.

Interaction matrices were visualized using Cytoscape 3.10.3 [43]. Shortest communication pathways were identified using Dijkstra’s algorithm [44], with particular emphasis on signal propagation between highly flexible loop regions and the catalytic core. Domain-level interaction networks were constructed by grouping residues into six RMSF-based dynamic regions defined for each enzyme.

## 5. Conclusions

In this study, we systematically investigated the temperature-dependent conformational dynamics and thermal adaptation mechanisms of WT-PETase, FAST-PETase, and LCC-ICCG by integrating long-timescale molecular dynamics simulations with free energy landscape analysis, rigidity analysis, and neural relational inference. Our comparative analyses demonstrated that the three PETases adopt fundamentally different thermal adaptation strategies, ranging from flexibility-driven regulation to rigidity stabilization and network-resilient communication, highlighting fundamentally different molecular solutions for preserving structural stability and dynamic coordination under thermal stress.

Overall, our findings support the proposed hypothesis that the enhanced thermostability of engineered PETases arises from a favorable balance between structural rigidity and conformational flexibility, accompanied by the preservation of long-range residue communication under thermal stress. These results demonstrate that enzyme thermostability cannot be explained solely by increased structural rigidity, but instead emerges from the coordinated regulation of structural stability, conformational dynamics, and allosteric communication. By integrating molecular dynamics simulations with network-based analyses, this study provides new mechanistic insights into the molecular basis of PETase thermal adaptation and establishes a theoretical framework for the rational engineering of efficient and thermostable PET-degrading enzymes for sustainable plastic recycling.

## Data Availability

The original contributions presented in this study are included in the article/Supplementary Material. Further inquiries can be directed to the corresponding authors.

## Supplementary Materials

The following supporting information can be downloaded at: https://www.mdpi.com/article/10.3390/ijms27146531/s1.
