# Supplementary material for: Temperature-Dependent Reorganization of Conformational Dynamics and Interaction Networks Underlies Thermostability in PET-Degrading Enzymes
Source: Int J Mol Sci. 2026 Jul 22;27(14):6531. doi: 10.3390/ijms27146531 (PMC13410083; doi:10.3390/ijms27146531)
Supplement: Supplementary file 1 [file ijms-27-06531-s001.zip › ijms-4394064-supplementary.pdf]

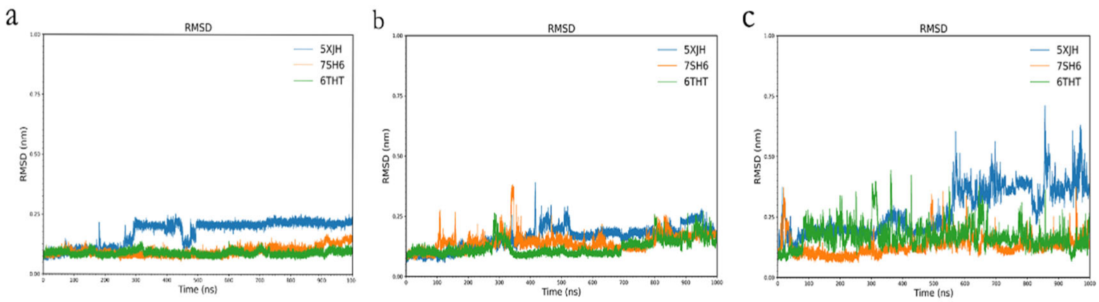

Figure S1. Backbone root mean square deviation (RMSD) profiles of PET-degrading enzymes during 1 μs molecular dynamics simulations. Panels (a–c) correspond to simulations performed at 30 °C, 50 °C, and 70 °C, respectively. The RMSD values were calculated relative to the initial structure. Different colors represent WT-PETase (5XJH), FAST-PETase (7S6H), and LCC-ICCG (6THT). The time evolution of RMSD reflects the temperature-dependent conformational stability of the three enzymes.

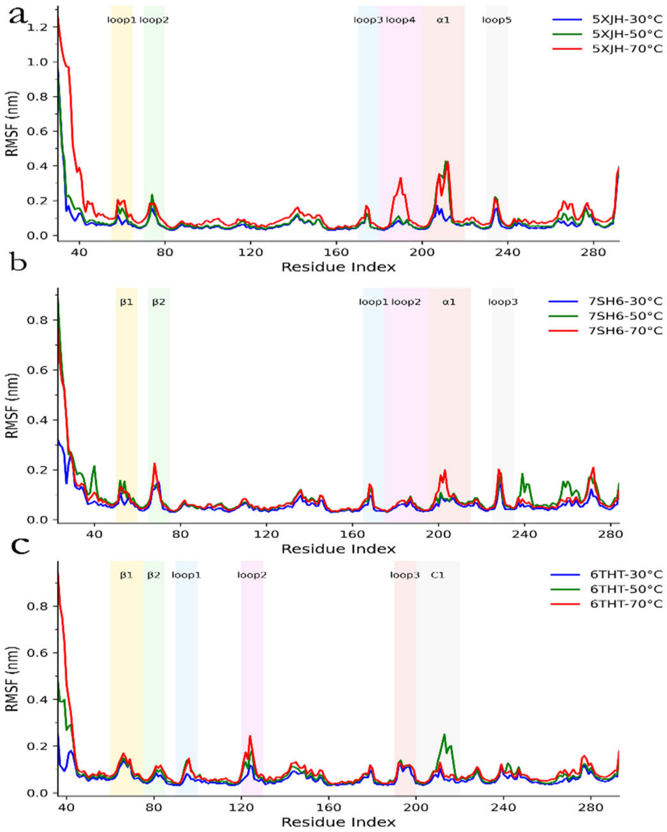

Figure S2. Temperature-dependent RMSF profiles of PET-degrading enzymes. Root-mean-square fluctuation (RMSF) profiles of Cα atoms for (a) WT-PETase (PDB: 5XJH), (b) FAST-PETase (PDB: 7SH6), and (c) LCC-ICCG (PDB: 6THT) at 30 °C (blue), 50 °C (green), and 70 °C (red). Shaded regions indicate key secondary-structure elements and functional segments, including β-strands, α-helices, and loop regions. The RMSF distributions reveal distinct temperature-dependent flexibility patterns among the three enzymes, highlighting differences in dynamic responses and thermal adaptation across structurally and functionally important regions.

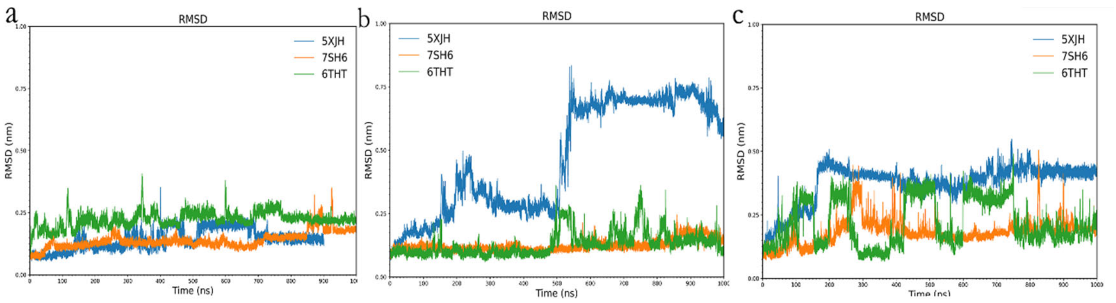

Figure S3. Root mean square deviation (RMSD) profiles obtained from independent replicate molecular dynamics simulations of WT-PETase (5XJH), FAST-PETase (7SH6), and LCC-ICCG (6THT) at (a) 303 K, (b) 323 K, and (c) 343 K. The replicate simulations exhibit overall RMSD trends consistent with those observed in the original simulations, confirming the reproducibility and robustness of the conformational behaviors.

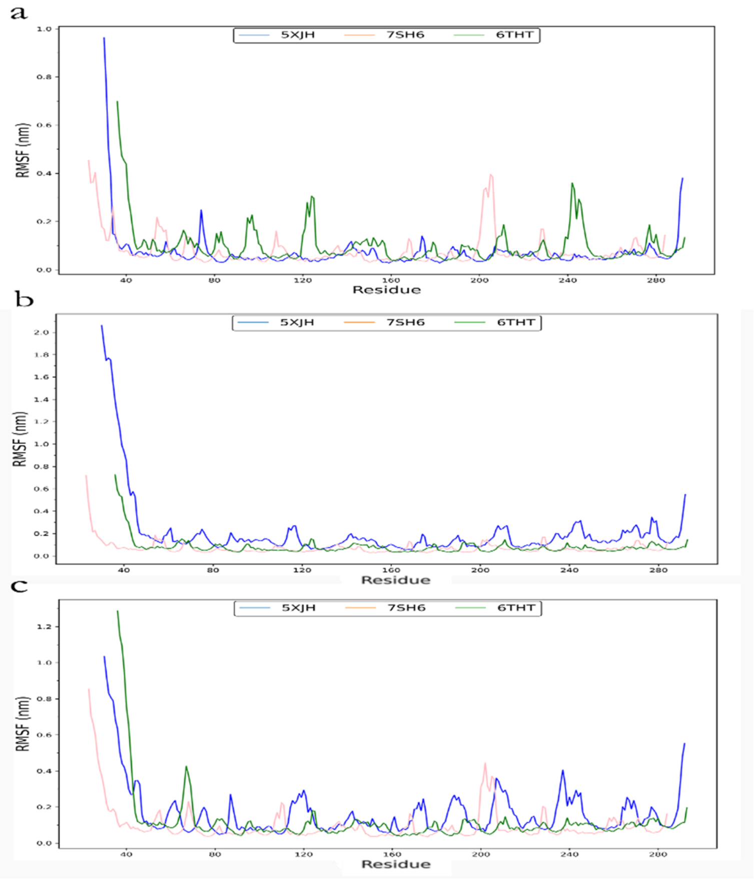

Figure S4. Root mean square fluctuation (RMSF) profiles obtained from independent replicate molecular dynamics simulations of WT-PETase (5XJH), FAST-PETase (7SH6), and LCC-ICCG (6THT) at (a) 303 K, (b) 323 K, and (c) 343 K. The replicate simulations reproduced the overall residue flexibility patterns observed in the original simulations, confirming the robustness and reproducibility of the temperature-dependent conformational dynamics.

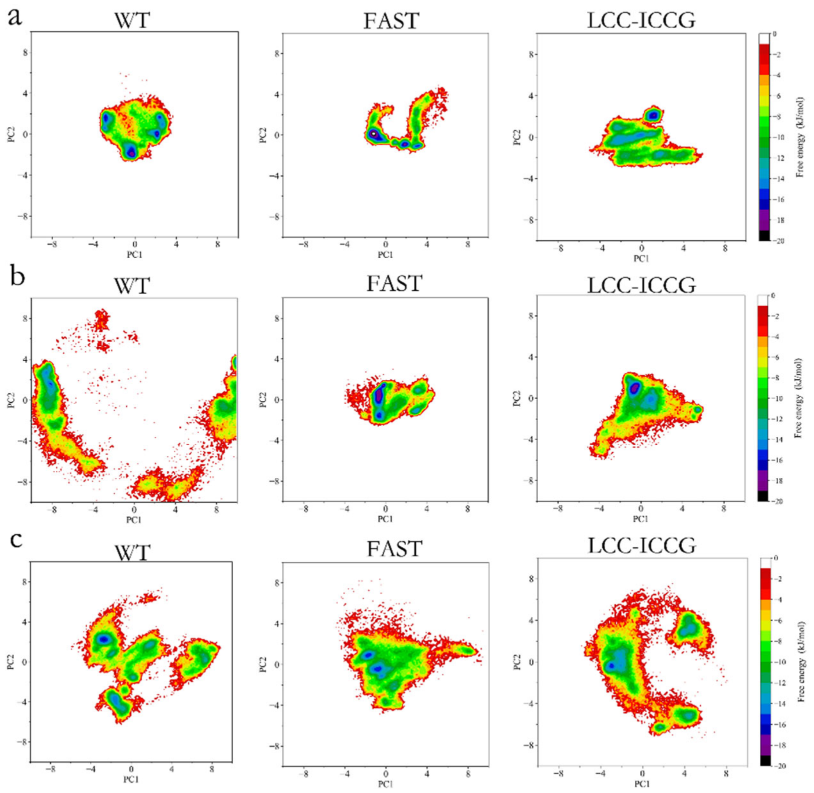

Figure S5. Free energy landscapes (FELs) obtained from the independent replicate molecular dynamics simulations of WT-PETase, FAST-PETase, and LCC-ICCG. The free energy landscapes were constructed by projecting the trajectories onto the first two principal components (PC1 and PC2). Panels (a–c) correspond to simulations performed at 303 K, 323 K, and 343 K, respectively. The color scale represents the relative free energy (kJ/mol), with blue indicating low-energy, highly populated conformational states and red indicating high-energy, less populated states. The replicate simulations reproduce the overall free energy distributions observed in the primary simulations, confirming the reproducibility of the temperature-dependent conformational landscapes.
